# Real-Time 3-Dimensional Ultrasound-Assisted Infraclavicular Brachial Plexus Catheter Placement: Implications of a New Technology

**DOI:** 10.1155/2010/208025

**Published:** 2010-06-01

**Authors:** Steven R. Clendenen, Christopher B. Robards, Nathan J. Clendenen, James E. Freidenstein, Roy A. Greengrass

**Affiliations:** ^1^Department of Anesthesiology, Mayo Clinic College of Medicine, Mayo Clinic, Jacksonville, FL 32224, USA; ^2^Royal College of Surgeons, 123 St. Stephens Green, Dublin 2, Ireland

## Abstract

*Background*. There are a variety of techniques for targeting placement of an infraclavicular blockade; these include eliciting paresthesias, nerve stimulation, and 2-dimensional (2D) ultrasound (US) guidance. Current 2D US allows direct visualization of a “flat” image of the advancing needle and neurovascular structures but without the ability to extensively analyze multidimensional data and allow for real-time manipulation. Three-dimensional (3D) ultrasonography has gained popularity and usefulness in many clinical specialties such as obstetrics and cardiology. We describe some of the potential clinical applications of 3D US in regional anesthesia. *Methods*. This case represents an infraclavicular catheter placement facilitated by 3D US, which demonstrates 360-degree spatial relationships of the entire anatomic region. *Results*. The block needle, peripheral nerve catheter, and local anesthetic diffusion were observed in multiple planes of view without manipulation of the US probe. *Conclusion*. Advantages of 3D US may include the ability to confirm correct needle and catheter placement prior to the injection of local anesthetic. The spread of local anesthetic along the length of the nerve can be easily observed while manipulating the 3D images in real-time by simply rotating the trackball on the US machine to provide additional information that cannot be identified with 2D US alone.

## 1. Introduction

Continuous infraclavicular brachial plexus block is a popular technique for postoperative analgesia following upper extremity surgery [1]. Nerve localization techniques used for the placement of peripheral nerve catheters include nerve stimulation [2] and 2-dimensional (2D) ultrasound (US) guidance [3, 4]. We describe a 3-dimensional (3D) US technique in which an X 7-2 matrix ultrasound probe (Philips IU-22, Andover, MA) was used for the placement of an infraclavicular nerve block, providing improved imaging of the advancing needle and nerve bundle with real-time manipulation of the US images.

## 2. Case Report

The Mayo Clinic Institutional Review Board approved review of the patient's medical record.

A 64-year-old man was scheduled for a right total elbow arthroplasty and consented for an infraclavicular brachial plexus nerve catheter for postoperative analgesia.

**Figure 1:**
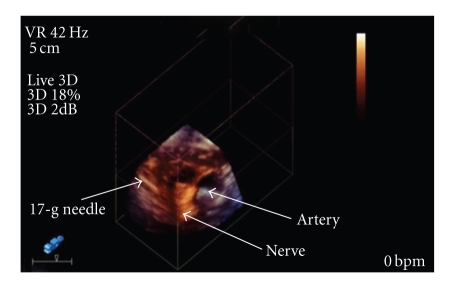
Oblique view of the needle at the posterior cord.

**Figure 2:**
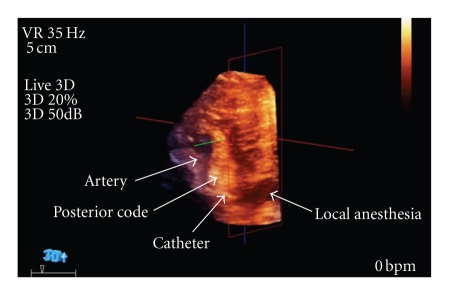
Oblique view of the nerve catheter inferior to the posterior cord.

The patient was positioned supine with his neck turned 45 degrees away from the side of the block, and the coracoid process was identified. After cleansing and draping the skin, a sterile sheath was applied to the US probe. The skin was anesthetized with 2% lidocaine and sterile US gel was applied. The 3D US probe was positioned 2 cm medially and 2 cm caudal to the coracoid process. A 17-gauge insulated 50-mm Arrow Tuohy needle (Arrow International; Reading, PA) connected to a nerve stimulator (standard for our institution set at pulse duration 0.3 ms, current 1.5 mA, frequency 2 Hz) was advanced in a saggittel plane toward the nerves with 3D US visualization (Figure 1). After correct positioning was corroborated by appropriate distal motor nerve response of the posterior cord, a 19-gauge stimulating catheter (Stimcath, Arrow International; Reading, PA) was advanced 5 cm past the needle tip under continuous stimulation. Local anesthesia was administered through the catheter and was observed tracking circumferentially around the nerve bundle as well as proximally and distally. This was viewed in multiple planes by rotation of the trackball on the US machine by an assistant which manipulates in real-time the 3D images. After injection of 30 cc of local anesthetic (ropivacaine 0.5%), the catheter was visualized lying across the nerve bundle (Figure 2).

The patient had a complete motor and sensory block in the radial, median, ulnar, and musculocutaneous nerve distributions prior to undergoing successful total elbow arthroplasty. An indwelling catheter with continuous infusion of dilute local anesthetic (ropivacaine 0.2%) managed postoperative pain with minimal oral narcotic supplementation. The catheter was discontinued on postoperative day 3 without complications.

## 3. Discussion

The early clinical application of 3D US began in the fields of echocardiography, obstetrics, and intravascular medicine. The limitation of 3D US technology was dictated by transducer development and the computing power of the ultrasound equipment which required image viewing to be performed at offline workstations. The advancement of US technology has seen an increase in the utilization of 3D US with the findings of a recent literature review of over 20 different categories of clinical applications. The current 3D transducers and US machines were not designed for regional anesthesia and not practical at this time for routine block placement. The advancement of technology has resulted in laptop 3D US machines along with transducers being developed with greater resolution with a potential use for regional anesthesia.

**Figure 3:**
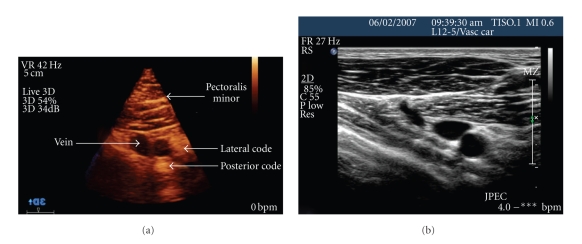
(a) Three-dimensional infraclavicular ultrasound image. (b) Two-dimensional infraclavicular ultrasound image.

The comparison between 2D and 3D US is analogous to the comparison of plain X-ray and computerized tomography. Figure 3 Two common means to obtain 3D images are by a mechanical or matrix ultrasound probe. The acquisition of 3D US images can be obtained by a mechanically swept transducer that is mounted inside a standard probe that moves through a known trajectory and obtains 2|D scans which are then converted into a 3D image. Unlike 2D US that acquires a planar image, the matrix array transducer utilizes more than 2,400 piezoelectric elements, which both transmit and receive data and acquires a direct 3D image signal. The processing power of the modern computer facilitates real-time acquisition of images, which are then displayed as 3D data sets in real time—the fourth dimension (4D). Advantages of 4D US is that the probe can be held stationary, the large band of US data can be manipulated, and real-time visualization of the needle can be accomplished in multiple planes, thus improving the nerve localization and helping avoid vascular structures. 

Current 2D US technology captures a planar (flat) image, in contrast with 4D US that acquires multiple planes of view simultaneously without having to reposition the US probe. The difficulty is tracking the needle in the large volume of information in the 4D mode. The X7-2 probe US technology with a touch of a button changes the standard 2D US view to the less familiar 4D view and a thick slice mode that enhances needle tracking. In this preliminary imaging study of a femoral nerve block, we were able to track the needle in thick slice mode and observe the nerve and local anesthetic spread in 4D [5]. This information enables multiple planes of view by manipulating the image without movement of the US probe. Unintentional probe movement during the performance of a block has been identified as an error in the hands of novices when using 2D US [6]. On 3D US, nerves appear as hyperechoic, cord-like structures when viewed on long axis; correct nerve identification can then be confirmed by eliciting appropriate motor response to nerve stimulation.

Current 2D images only provide a “doughnut” sign of spread of local anesthesia in one plane in contrast to 3D imaging which will provide 360-degree data set on the distribution of the local anesthesia around the nerves. One of the early descriptions of real time 3D US utilized the matrix array probe (X 3-1 Philips Medical Systems; Andover, MA) to observe the distribution of local anesthetic in multiple planes during injection of a popliteal sciatic nerve catheter [7]. The X 3-1 probe has an interrogation frequency of 1–3 MHz, which provides suboptimal resolution for regional anesthesia applications. The next generation matrix probe, the X 7-2, (designed for 3D US of the pediatric heart) has a higher resolution of 2–7 MHz which allows better visualization of nerves and was recently used to identify aberrant popliteal sciatic nerve bifurcation [8]. 

Additionally, higher 3D US frequency of the X 7-2 probe allows for detailed imaging of superficial nerve bundles in relation to anatomical landmarks, and the image can be manipulated in real time to provide further evidence of a correctly positioned needle placement and spread of local anesthetic. This approach is especially useful in nerve blocks involving the brachial plexus and any area of anatomy that has a dense population of neurovascular structures and individual anatomic variations. 

The 3D US imaging can provide information of the entire anatomic region including 360-degree spatial relationships of adjacent structures. A recent article employing the X 7-2 probe demonstrated the influence of arterial pulsation on the spread of local anesthetics during an axillary brachial plexus block [9]. The X 7-2 probe on the new iU22 ultrasound system (Philips Medical Systems; Andover, MA) captures the standard 2D US view; however, real-time 3D imaging can be visualized on demand, allowing the practitioner to compare the same anatomic structures in both 2D and 3D (sonoanatomy).

## 4. Conclusions

The utilization of 3D US for regional anesthesia is in its infancy and further investigations and studies are needed. The 3D US technology is being developed and manufactured for a multitude of clinical practice in medicine, and with these advancements there may be some application into the field of regional anesthesia. Advantages of 3D US may include the ability to monitor correct needle placement and spread of local anesthetic along the nerve and better identify adjacent unwanted targets (pleura and blood vessels) while manipulating the images in real-time to provide additional information that cannot be identified with 2D US alone. A recent editorial proposed questions on the ideal pattern of local anesthesia spread for a safe and effective block and 3D US may be a tool in the future for this research [10].

Further development of 3D US probes with even higher frequency capabilities is needed to further enhance image quality, while improved linear probe designs would be helpful in tracking the needle during performance of the block [11].
